# User Experience and Socioemotional Evaluation of Virtual Therapist Character Types in a Simulated Virtual Reality Exposure Therapy: Quantitative Study

**DOI:** 10.2196/81537

**Published:** 2026-08-07

**Authors:** Michelle Celina Hallmann, Nicolina Laura Peperkorn, Francesco Vona, Youssef Shiban, Jan-Niklas Voigt-Antons

**Affiliations:** 1 Immersive Reality Lab, Hamm-Lippstadt University of Applied Sciences, Marker-Allee 76-78, Hamm, 59063, Germany, 49 23818789 ext 914; 2 Department of Clinical Psychology, PFH Private University of Applied Sciences, Göttingen, Germany

**Keywords:** embodied conversational agent, virtual character, virtual therapist, Uncanny Valley, virtual reality exposure therapy

## Abstract

**Background:**

Virtual reality exposure therapy (VRET) has emerged as an effective treatment for individuals with specific phobias, particularly for those unable to access traditional exposure therapy. However, several aspects of VRET applications still require further investigation, including the role of embodied conversational agents as virtual therapists.

**Objective:**

This study aims to investigate how the realism level and species of a virtual therapist influence user experience and perceived socioemotional relationship skills in a VRET intervention for dog phobia.

**Methods:**

We conducted a quantitative study that examined user experience and perceived socioemotional relationship skills of virtual therapist character types, where participants were recruited from a convenience sample and were not required to have a dog phobia. In 4 simulated VRET interventions, participants interacted with 4 virtual therapists through a role-play dialogue (3.5 minutes per scenario), followed by an exposure scenario with a dog. The 4 virtual character types included a realistic adult woman, a stylized adult woman, a stylized cat, and a stylized robot. We collected quantitative data through standardized questionnaires assessing user experience and presence, cybersickness, trust, and willingness to share information, and an exploratory, theory-informed questionnaire assessing the virtual therapist’s socioemotional relationship skills.

**Results:**

A total of 40 participants completed the experiment. The realistic human scored significantly lower in hedonic quality than the stylized human (*z*=2.53; *P*=.01; *P*_adj_=.02; rbb=−0.54), the robot (*z*=2.726; *P*=.006; *P*_adj_=.01; rbb=−0.56), and the cat (*z*=3.772; *P*<.001; *P*_adj_=.004; rbb=−0.81). Additionally, the cat scored higher in hedonic quality than the stylized human (*z*=2.528; *P*=.02; *P*_adj_=.02; rbb=−0.52). While no significant differences were found among the virtual character types regarding trust or willingness to share information, the therapeutic relationship skills of the robot were rated significantly lower than those of the stylized human (*z*=−2.315; *P*=.02; *P*_adj_=.02; rbb=−0.42). However, when asked to indicate a preferred virtual therapist, 30% (12/40) of the participants chose the robot, 27.5% (11/40) the stylized human, 25% (10/40) the cat, and 17.5% (7/40) the realistic human, making the latter the least favored preferred option in our study sample.

**Conclusions:**

The realistic human was the least preferred virtual therapist, whereas the stylized human received more favorable evaluations. This finding suggests that increased visual realism does not necessarily result in more positive perceptions of virtual therapists and may, in some cases, lead to less favorable user responses. The cat’s higher hedonic quality and the robot’s popularity suggest that user perceptions may vary across different virtual therapist embodiments. These findings underscore the need for further research on virtual therapist design in VRET.

## Introduction

### Background

Specific phobias are among the most common mental disorders, with lifetime prevalence estimates ranging from 7.7% to 12.5% among adults [1]. Individuals with specific phobias experience intense, irrational fear responses and exhibit avoidance behavior toward a specific object or situation [2]. Exposure therapy, particularly in vivo treatment, is the preferred method for helping individuals replace maladaptive fear responses with adaptive reactions [3]. Exposure therapy for specific phobias typically begins with psychoeducation, during which patients learn how phobias develop and persist through avoidance behavior, and which fears are rational or irrational [4,5]. Patients are then systematically confronted with the feared object or situation, enabling cognitive restructuring by reducing maladaptive behaviors and replacing irrational fear responses with more adaptive interpretations [2,4,5]. Despite the proven effectiveness of exposure therapy [6], fewer than 8% of individuals with specific phobias seek treatment [7]. Barriers include the unpredictability of exposure, challenges in implementing therapy, and a shortage of mental health care providers [6,8,9].

An alternative approach, known as in virtuo treatment, uses virtual reality (VR) to address many of these barriers. VR enables users to experience computer-generated environments that can evoke a strong sense of presence or "sense of being there," which is commonly attributed to the replacement of real-world sensory input with computer-generated stimuli [10]. Through this effect, the user cognitively knows that the world around them is unreal, yet experiences a perceptual illusion that makes the brain and the body automatically react to sensory cues [11]. This characteristic of VR allows patients to repeatedly confront realistic phobic stimuli in a manner comparable with traditional in vivo exposure conducted in real-life settings. But in contrast to real-world exposure, virtual reality exposure therapy (VRET) allows therapists to precisely control and gradually adjust exposure intensity according to individual patient needs, offering high repeatability and flexibility while maintaining a sense of safety for the patient [12]. This controlled environment may reduce barriers to treatment and encourage engagement with exposure exercises that might otherwise be avoided in real-life situations. Several meta-analyses have reported greater patient willingness to engage in VRET compared with in vivo exposure [13-15], which has frequently been attributed to the perceived safety and controllability of the virtual environment. The findings of a more recent meta-analysis on VRET for specific phobias, which reported no significant differences between VRET and traditional in vivo exposure at posttreatment or follow-up [16], together with the inclusion of VRET in the American Psychological Association guidelines for empirically supported treatments, demonstrate the effectiveness of VRET for specific phobias [17].

Furthermore, VRET can expand access to treatment for individuals who face barriers to traditional methods, such as limited facilities, practitioner shortages, cultural disparities, or life circumstances [18]. One possible approach is the use of automated interventions, also referred to as guided self-help [19], which have recently been shown to achieve substantial symptom reductions [20]. However, findings from internet-based mental health interventions have suggested that maintaining treatment adherence can be challenging, particularly in unguided approaches [21]. This raises the question of how therapeutic guidance, psychoeducation, and emotional support can be delivered in the absence of a physically present therapist, for example, through virtual therapists that serve as a functional analog to human therapists during exposure exercises [22,23].

To date, several studies have explored automated VRET interventions incorporating virtual therapists, coaches, or health agents to provide guidance, psychoeducation, and user support throughout the treatment process [19,20,24,25]. Although these studies primarily focused on treatment efficacy and the implementation of exposure procedures, later work increasingly evaluated users’ experiences and perceptions of virtual therapists and automated VRET systems [26-28]. A recent systematic review highlighted the increasing use of virtual humans in VR mental health research and the diversity of their implementations [29].

The implementation of these virtual therapists varied substantially across studies. Donker et al [24] incorporated a 2D animated avatar named *Tara* to deliver background information on phobias via a smartphone app. Miloff et al [25] incorporated voice-over psychoeducation, treatment instruction, and guidance through the exposure tasks in a VRET application for spider phobia primarily. Freeman et al [19], Lindner et al [20], and Wei et al [28] incorporated embodied conversational agents (ECAs) into automated VRET applications to provide psychoeducation, therapeutic guidance, and user support. While Lindner et al [20] used an animated holographic virtual therapist primarily for introductory psychoeducation, Wei et al [28] implemented an embodied virtual coach that conducted interactive psychoeducational consultations. Freeman et al [19] further extended this approach by using an embodied virtual coach not only for psychoeducation and assessment but also to guide participants throughout exposure exercises. ECAs are virtual agents designed to engage in interactive dialogues with users [30] and possess a virtual embodiment, enabling them to simulate real-world social interactions. Such interactions may enhance treatment adherence and efficacy through, that is, further immersion into the therapeutic context and a more natural way of conveying psychoeducation and reinforcing progress [31]. Consequently, the implementation of virtual therapists and ECAs has already been explored in various anxiety- and phobia-related applications beyond clinical trials of automated VRET interventions [32].

Given that the therapist-patient relationship can influence therapy outcomes [33] and human-agent interactions can evoke social and emotional responses similar to human-human interactions [34,35], examining how various aspects of ECA design impact users in clinical settings is crucial. Prior research suggests that the representation and embodiment of virtual interaction partners can influence users’ sense of social presence and interpersonal engagement within immersive environments [36,37]. Social presence, defined as the sense of “being with another” [38], has been associated with factors such as empathy, involvement, and affective responses during social interactions [39]. Furthermore, trust and perceived listening behavior have been identified as important characteristics of virtual human interactions that can influence users’ willingness to disclose personal information [40]. In therapeutic settings, these interpersonal and social interaction factors may further contribute to the development of therapeutic alliance between users and virtual therapists [41]. Factors such as visual appearance, social cues, and interactivity may therefore shape how users perceive and interact with virtual therapists during VRET interventions.

A scoping review by Provoost et al [42] further highlighted the importance and complexity of determining the optimal appearance of ECAs in a clinical context to enhance treatment success. As 1 key aspect of ECAs, appearance can be broken down into subfeatures such as degree of realism, species, gender, or clothing [43]. Previous research has examined how visual realism influences users’ perceptions of virtual humans in social and conversational contexts. However, findings remain mixed, with studies suggesting that increased realism may affect aspects such as social presence, trust, and engagement, while others report little or no effect on these outcomes [36,44]. Beyond realism, the embodiment form of an artificial agent may influence how users perceive and interact with it. Previous research suggests that appearance establishes social expectations and can bias interactions, with human-, animal-, and machine-like embodiments potentially eliciting different responses [45]. Nevertheless, a recent systematic review revealed that extended reality–based ECA research has overwhelmingly focused on human-like embodiments, with only a small number of studies investigating robotic agents and virtually none exploring animal-like embodiments [46]. Consequently, little is known about how different embodiment species influence users’ perceptions and experiences, and even less is known about their role in therapeutic extended reality applications such as VRET. Therefore, this study focuses on realism and species as 2 appearance dimensions that may shape users’ perceptions of virtual therapists in VRET.

### Degree of Realism of an ECA

A literature review by ter Stal et al [47] on the effects of ECAs design features in eHealth found mixed results regarding the impact of realism on user perception. While stylized ECAs have been rated more positively on friendliness, several studies indicated a preference for more realistic ECAs. However, findings regarding the optimal rendering style for ECAs varied, with significant differences in study design and participant demographics. The authors emphasized the need for further research to understand better how different rendering styles influence user experience.

McDonnell et al [48] explored how different rendering styles influence how people perceive animated virtual humans. They developed 10 virtual characters, ranging from abstract to realistic styles, and found that motion anomalies were perceived as less unpleasant in stylized virtual humans than in realistic virtual humans. This finding aligns with the Uncanny Valley effect described by Mori et al [49], which suggests that as a robot becomes more human-like, affinity toward it increases until it reaches a threshold where slight imperfections cause unease. Beyond this point, as realism continues to improve, affinity rises again. Research over the past decades has shown that Mori’s observations extend to virtual characters as well [50]. Consequently, the Uncanny Valley effect must be carefully considered when designing ECAs for virtual environments to ensure positive user engagement and acceptance.

### Species of an ECA

ECAs can take various forms beyond human representations, including animalistic, robotic, or fantastical creatures. A review by Mitrut et al [32] showed that virtual therapists and similar ECAs have been embodied in a wide range of forms, including male and female human avatars, photographic representations of clinicians, fantasy characters, and virtual animal characters. This diversity suggests that no consensus has yet emerged regarding the most suitable embodiment for virtual therapists. Animalistic ECAs have been studied in different contexts as digital conversation partners. For example, Chi et al [51] tested a tablet-based ECA system as a digital pet in the homes of 10 older adults. Although participants desired more extensive conversational capabilities, most developed a sense of companionship with the digital pet and appreciated its social support, health information, instant assistance, and entertainment features. A quantitative follow-up study by Bott et al [52] using the same pet avatar with older adults in a hospital found that the pet avatar helped alleviate symptoms of delirium and loneliness.

Although research on the Uncanny Valley effect in nonhuman virtual characters is limited [53], studies on the impact of realism in virtual animals suggest that an Uncanny Valley effect also exists for animalistic characters [53,54]. Notably, Sierra Rativa et al [54] investigated virtual pets with varying degrees of realism, examining dimensions such as familiarity, naturalness, attractiveness, and animateness. Their results showed that movement amplified the Uncanny Valley effect, while morbid features led to more negative perceptions of virtual pandas. Furthermore, differences between expert evaluations and participant responses regarding animal likeness underscored the nuanced ways virtual animals are perceived across various dimensions.

### This Study

Previous work on VRET applications, including virtual therapists for psychoeducation or guidance, has rarely examined how appearance-related design dimensions influence users’ socioemotional responses to these agents. While voice, behavior, and interactivity are also important design dimensions, realism and species were selected because they directly shape the visual embodiment of virtual therapists. Although realism has received considerable attention in prior research, findings remain inconclusive, whereas embodiment species have received comparatively little attention. At the same time, most prior work has examined these design dimensions in general interaction settings rather than therapeutic exposure scenarios, in which virtual therapists act as social interaction partners, providing psychoeducation and guidance prior to exposure. To address this gap, we selected dog phobia as an example application, as specific phobias are among the most established applications of VRET and provide a suitable context for investigating how users perceive virtual therapists in therapeutic exposure scenarios. Furthermore, dog phobia is particularly suitable for VRET, as encounters with dogs can be simulated in immersive virtual environments while retaining key characteristics of real-world exposure situations. By implementing interactive psychoeducational dialogues prior to exposure, we examined how ECA embodiments varying in realism and species influenced users’ experiences and perceptions of a virtual therapist in a simulated VRET intervention for specific phobias. Our findings aim to assist interdisciplinary teams in making informed decisions when integrating ECAs into research and clinical applications.

## Methods

### Experimental Design

We developed a VRET application for dog phobia and conducted a quantitative study in which each participant interacted with 4 distinct virtual therapist characters across 4 different interventions. The interventions varied in their psychoeducational content and exposure scenarios to maintain participant engagement. As the psychoeducational content was designed to build upon content presented in previous interventions, the intervention scenarios followed a fixed order for all participants, while virtual character assignment to sessions was randomized. Each intervention consisted of 2 main components: a virtual therapist providing psychological education and exposure to a virtual dog in the controlled VR environment. We measured several dependent variables related to the virtual characters, including user experience, therapeutic relationship skills, and trust as perceived by the participants. Additionally, we defined 4 different intervention scenarios as independent variables to analyze their effects on user experience, cybersickness, and presence. Our goal was to assess the potential of the application for follow-up studies with patients and evaluate the comparability of the virtual therapists across different intervention scenarios.

To ensure that participants could meaningfully assess the virtual therapist within the given context, we designed interactive dialogues between the participant and the virtual therapist. A Wizard-of-Oz approach was used, in which a human operator simulated the virtual therapist’s responses [55]. This approach was chosen to ensure consistent therapeutic content and interaction quality across all virtual therapist embodiments while maintaining a natural conversational experience. The dialogue structure was predefined, with participants verbally responding to questions posed by the virtual therapist. Depending on the participant’s response, the operator triggered corresponding scripted responses in real time. By relying on predefined dialogue paths, variability in the delivered content was minimized and experimental control was maintained across conditions. The same researcher operated the system throughout all study sessions to further ensure consistency in dialogue delivery.

### Recruitment

This study is an initial exploratory evaluation of different virtual character types in a VRET application, aiming to generate insights for further interventions with patients with dog phobia. Subsequently, 40 participants from a convenience sample of healthy adult volunteers were recruited via the university’s student portal and on-campus notices. No specific inclusion criteria were required for participation, and each participant received a €15 (€1=US $1.12 as of July 17, 2023) compensation. The study took place in our Immersive Reality laboratory over a period of 2 weeks in July 2023.

### Virtual Character Types

**Figure 1.**
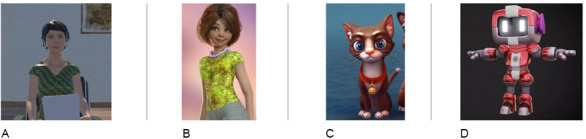
Virtual character types used in the study (from left to right): (A) realistic adult woman, (B) stylized adult woman, (C) stylized cat, and (D) stylized robot.

The 4 virtual character types chosen for the study were a realistic adult woman, a stylized adult woman, a stylized cat, and a stylized robot (Figure 1). The realistic and stylized human characters represented 2 levels of visual realism while maintaining the same species, gender, and clothing to facilitate comparison. The stylized cat was included to investigate an animal-like embodiment, whereas the stylized robot represented a machine-like embodiment. Together, these character types were selected to investigate how variations in realism and species influence users’ perceptions of virtual therapists within a simulated VRET intervention.

To enhance realism, we implemented lip synchronization and eye movement for the first 3 characters using the Unity SALSA Lip Sync Suite package (Crazy Minnow Studio). The 3D models for the realistic and stylized humans were sourced from Daz3D (Daz 3D), while the cat and robot models were obtained from the Unity Asset Store.

### Stimulus Material

Participants used a Meta Quest 2 VR headset during the experiment. They had no avatar representation, remained seated throughout the psychological education phase, and stood up for the exposure phase. The application was developed using Unity 3D (Unity Technologies), Daz Studio (Daz 3D), Visual Studio (Microsoft), and Audacity (The Audacity Team). We created 2 separate environments: 1 for the psychological education and 1 for the exposure.

A specialist wrote a separate dialogue for each intervention based on the psychological education principles related to specific phobias and exposure therapy. We prerecorded the virtual therapists’ dialogues using a female voice, ensuring consistency with the 2 humanoid virtual therapists. The dialogues included interactive questions, and the experimenter could trigger appropriate responses based on participant input. Participants went through these interactive dialogues at their own pace, which typically took around 3.5 minutes.

**Figure 2.**
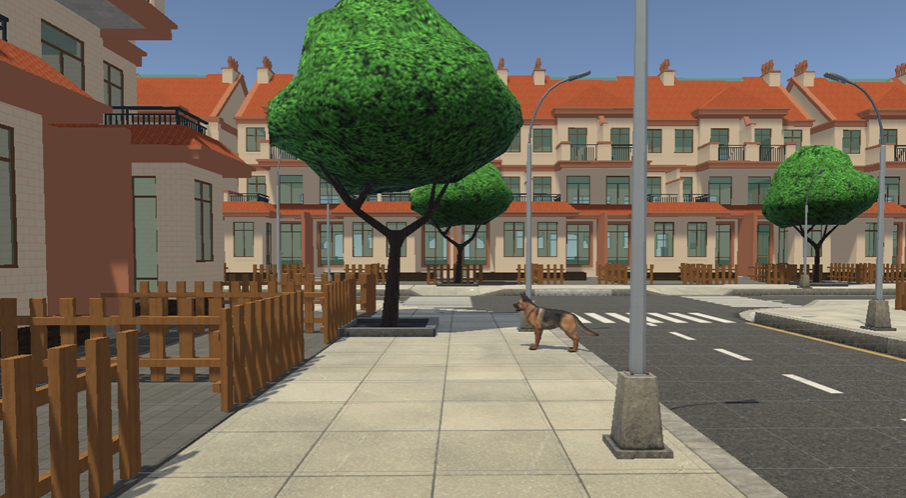
Exposure scene.

During the exposure phase, participants faced a shepherd dog standing, sitting, or lying on a sidewalk in an urban neighborhood (Figure 2). We set up 4 different exposure scenarios with varying walking directions and typical nonaggressive behaviors by the dog to keep participants engaged during the experiment. The implemented behaviors were intended to reflect typical everyday encounters with dogs while remaining comparable across the different exposure scenarios. After 55 seconds, the dog entered an idle state, meaning no further active behaviors were performed. Participants were then asked to remove the headset, resulting in approximately 1 minute of exposure per scenario. To ensure comparability between the exposure scenarios, the dog was positioned at a similar distance from the participant in all intervention scenarios. Because the content of the exposure scenarios was similar, they were conducted in the same order for all participants, with the therapist randomized in each scenario.

The experimenter controlled the intervention via a web app. The web app displayed the participant’s camera view, allowing the experimenter to trigger conversation content by clicking interface buttons. A local web server connected the web app with the Unity application running in the Unity Editor.

### Measures

An overview of the independent and dependent variables and the questionnaires used can be found in Table 1.

**Table 1.** Questionnaires used in the study.

Questionnaire	Items	Dependent variables	Independent variables
Sociodemographic questionnaire	7	Age, gender, occupation, VR experience, presence of anxiety disorder or phobia, and presence of uncorrected sensory impairment	N/A^a^
Socioemotional questionnaire	48	Therapeutic relationship skills, emotions in therapeutic work	Virtual character type
Virtual therapist–patient relationship	2	Trust, willingness to share information	Virtual character type
User Experience Questionnaire Short Version	8	Pragmatic quality, hedonic quality, and overall quality	Virtual character type, intervention scenario
Affinity for technological interaction scale	9	Affinity for technological interaction	N/A^a^
Igroup presence questionnaire	1	Presence	Virtual character type, intervention scenario
Short cybersickness questionnaire	1	Cybersickness	Intervention scenario
Postquestionnaire	4	Preference for virtual character type, empathy for dog phobic	Virtual character type

a N/A: not applicable.

#### Therapeutic Relationship Skills and Emotions in Therapy Work

The socioemotional questionnaire used in this study was developed in a theory-informed manner based on the systematic review by Heinonen and Nissen-Lie from 2019 [56], which summarizes professional and interpersonal therapist characteristics associated with therapeutic effectiveness. The selected items were adapted for use in virtual therapist interactions during VR exposure therapy to capture participants’ perceptions of different virtual character types. As no standardized instrument specifically addressing these interaction characteristics in immersive VR therapeutic settings was available, a custom exploratory questionnaire was considered appropriate for this study’s aims.

The questionnaire consists of 3 sections. The first 2 sections assess the therapist’s relational skills, while the third section evaluates emotions in therapeutic work. The questionnaire was inspired by the “Questionnaire of Supervision, Intervision, and Self-awareness” from the Medical School Hamburg and the State Chamber of Psychotherapists Baden-Württemberg and the “Development of Psychotherapists Common Core Questionnaire” by Orlinsky et al [57]. Responses were recorded on a scale from 0 (do not agree at all) to 3 (completely agree), with an option for “kA” (no response).

#### Virtual Therapist–Patient Relationship

To adapt the questionnaire to a virtual therapy context, 2 additional questions were included: first regarding the willingness to share information with the virtual therapist, adapted from DeVault et al [58], and the second on the trust in the virtual therapist, based on the importance of trust in user interactions [59]. Both items were rated on a 5-point scale (1=not at all, 5=absolutely).

#### User Experience

As each intervention consisted of a psychoeducational dialogue with the virtual therapist followed by an exposure scenario, the User Experience Questionnaire Short Version (UEQ-S) [60] was administered after each intervention to assess the overall user experience. Because virtual therapist assignment was randomized across intervention scenarios, UEQ-S ratings were later analyzed with respect to both the virtual therapist embodiment and the intervention scenario. The UEQ-S uses a 7-point scale, with positive terms alternating on the left and the right.

#### Affinity for Technological Interaction

The participants’ technical affinity was assessed using the Affinity for Technology Interaction (ATI) scale [61]. This scale measures an individual’s propensity to engage with technological products, a factor that could significantly affect user experience. The questionnaire consists of 9 items on a 7-point Likert scale, ranging from 1 (completely disagree) to 6 (completely agree).

#### Presence

Effective virtual exposure therapy requires a natural and realistic environment for immersion [62]. To assess the sense of presence in the virtual environment, we used the “Igroup Presence Questionnaire” [63], specifically focusing on spatial presence. The scale ranges from 1 (no impression of being there) to 7 (strong impression of being there).

#### Cybersickness

Given the potential for cybersickness in VR applications [64], participants were asked about dizziness or nausea experienced during the intervention scenario. The scale ranges from 1 (no dizziness or nausea) to 5 (severe dizziness or nausea).

#### Postquestionnaire

After completing the experiment, participants indicated their preferred virtual therapist and provided justifications for their choice. These open-ended responses were collected to provide additional contextual information regarding participants’ preferences and were not subjected to formal qualitative analysis. They also assessed their ability to empathize with a dog-phobic individual during the simulation on a 7-point Likert scale (1=not at all, 7=very well). Participants could also provide qualitative feedback on their overall experience in an open-ended question.

### Procedure

The experiments were conducted in a university laboratory, ensuring a consistent and controlled environment throughout the study. At the start of each experiment, participants were given information about the study and asked to complete an informed consent form. They then filled out the sociodemographic questionnaire and the ATI scale. Afterward, participants were briefed on dog phobia and VRET to familiarize themselves with the experimental setting. Any questions from participants were addressed at this stage, and a brief text was provided to help immerse them in the role of a dog-phobic individual and encourage engagement during the interactions with the virtual therapists.

While participants read the provided information, the experimenter established the experimental conditions for the first intervention scenario. Participants were then instructed to sit in a chair and wear the VR headset. Once the experimenter triggered the “Start” button in the web app, the virtual therapist for that intervention appeared, and the dialogue between the virtual therapist and the participant began. When the dialogue ended, participants were instructed to stand up as the scene transitioned to the exposure environment.

After the exposure ended and participants removed the VR headset, they were given the socioemotional questionnaire, the virtual therapist–patient relationship questionnaire, the UEQ-S, the spatial presence item of the Igroup Presence Questionnaire, and the short cybersickness questionnaire. Once they had completed the questionnaires, the next intervention scenario began with another virtual character type embodying the therapist. This process was repeated for all 4 intervention scenarios. After the fourth intervention scenario, participants completed the postquestionnaire.

### Ethical Considerations

The planning, conduct, and reporting of the study were in accordance with the World Medical Association Declaration of Helsinki. All procedures were performed in compliance with relevant laws and institutional guidelines and were approved by the local ethics committee on July 13, 2023 (reference number EL20230701). The privacy rights of human subjects have been observed and informed consent was obtained from all participants before the start of the experiment.

### Statistical Analysis

Data were analyzed using SPSS (version 29; IBM Corp). Descriptive statistics are reported as means and standard deviations, consistent with the UEQ-S’s recommended reporting and with common practice in prior VR and human-computer interaction research using comparable questionnaire scales. Because the study used a repeated-measures design, analysis of significant differences was conducted using Friedman ANOVA. When a Friedman test indicated a significant difference, post hoc tests were conducted using the Wilcoxon signed-rank test. All statistical tests were 2-sided, and a significance level of α=.05 was applied. Effect sizes were reported as Kendall coefficient of concordance (Kendall *W*) for Friedman tests and matched-pairs rank-biserial correlations (rrb) for Wilcoxon signed-rank tests. The Benjamini-Hochberg correction was applied to adjust the *P* values obtained from the Wilcoxon signed-rank tests to account for multiple comparisons. This adjustment controlled for type I errors, reducing the likelihood of false-positive results. UEQ-S and presence scores were analyzed across intervention scenarios and virtual character types. Cybersickness scores were analyzed across intervention scenarios only. Scores of the socioemotional questionnaire and the virtual therapist–patient relationship questionnaire were analyzed across virtual character types. To evaluate the UEQ-S in comparison with the user experience of other systems, we used the official UEQ data analysis tool [60]. Open-ended responses were considered descriptively and used to contextualize the quantitative findings.

## Results

### Participant Characteristics

The study was conducted with 40 participants over 2 weeks. Participants’ ages ranged from 19 to 37 (mean 24.9, SD 3.86) years, with 25 (62.5%) participants identifying as male, 13 (32.5%) as female, and 2 (5%) as nonbinary. Out of 40 participants, 31 (77.5%) participants reported being students, 5 (12.5%) reported being research assistants, and 4 (10%) reported not working in a research-related field. Out of 40 participants, 34 (85%) participants had previous experience with VR, 15 (37.5%) participants reported having an anxiety disorder or phobia, 24 (60%) participants reported having no anxiety disorder or phobia, and 1 (2.5%) person declined to provide this information. The average ATI was mean 4.58 (SD 0.58).

### User Experience, Presence, and Cybersickness Across Intervention Scenarios

Table 2 presents the means, SD, and Friedman ANOVA results for user experience, presence, and cybersickness across the intervention scenarios. Analysis using Friedman ANOVA revealed no statistically significant differences in pragmatic, hedonic, or overall quality (UEQ-S scores) across the intervention scenarios. Similarly, no significant differences were found in the presence or cybersickness scores across the scenarios.

**Table 2.** Descriptive statistics and Friedman ANOVA results of user experience, cybersickness, and presence scores across intervention scenarios.

Dependent value	Scenario 1, mean (SD)		Scenario 2, mean (SD)		Scenario 3, mean (SD)		Scenario 4, mean (SD)		Chi-square (*df*)	*P* value	Kendall *W*
User experience											
Pragmatic quality	1.93 (0.73)		1.72 (1.02)		1.74 (0.995)		1.74 (1.15)		1.19 (3)	.76	0.010
Hedonic quality	1.5 (1.095)		1.56 (1.220		1.56 (1.17)		1.67 (1.18)		3.93 (3)	.27	0.033
Overall quality	1.72 (0.84)		1.64 (0.99)		1.65 (0.96)		1.71 (1.06)		0.67 (3)	.87	0.006
Presence	5.23 (1.1)		5.3 (1.22)		5.1 (1.41)		5.3 (1.44)		2.92 (3)	.40	0.024
Cybersickness	1.38 (0.67)		1.33 (0.7)		1.4 (0.84)		1.45 (0.81)		0.76 (3)	.86	0.006

### User Experience Across Virtual Character Types

**Figure 3.**
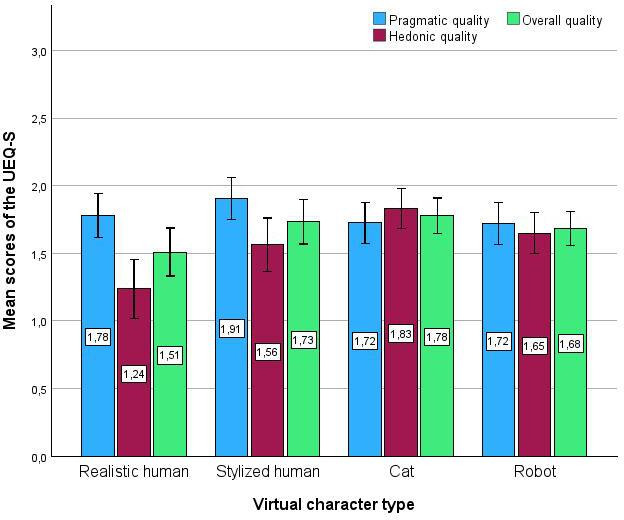
Mean scores of the UEQ-S across the therapists (blue: pragmatic quality, red: hedonic quality, green: overall quality; error bars show standard errors). UEQ-S: User Experience Questionnaire Short Version.

Table 3 provides the means, SD, and Friedman ANOVA results for user experience, presence, and cybersickness across the intervention scenarios. The means and SDs of the UEQ-S are also presented in Figure 3 for a better visual overview. As illustrated in Figure 3, differences between the virtual character types were primarily observed in hedonic quality, whereas pragmatic and overall quality ratings were relatively similar across characters. In particular, the realistic human received the lowest hedonic quality ratings, while the cat received the highest. As shown in Table 3, Friedman ANOVA did not reveal statistically significant differences in the pragmatic or overall quality across the virtual character types. However, a statistically significant difference was observed in the hedonic quality. Subsequent post hoc Wilcoxon signed-rank tests indicated that the hedonic quality of the realistic human was significantly lower than that of the other virtual character types. Specifically, comparisons showed inferior hedonic quality for the realistic human compared with the stylized human (*z*=2.53; *P*=.01; *P*adj=.02; rrb=−0.54), the robot (*z*=2.726; *P*=.006; *P*adj=.012; rrb=−0.56), and the cat (*z*=3.772; *P*<.001; *P*adj=.004; rrb=−0.81). Additionally, a significant difference in hedonic quality was observed between the stylized human and the cat, with the cat demonstrating higher hedonic quality (*z*=2.528; *P*=.02; *P*adj=.02; rrb=−0.52).

**Table 3.** Descriptive statistics and Friedman ANOVA results of user experience and socioemotional questionnaire scores across intervention scenarios.

Dependent value	Realistic human, mean (SD)		Stylized human, mean (SD)		Cat, mean (SD)		Robot, mean (SD)		Chi-square (*df*)	*P* value	Kendall *W*
User experience											
Pragmatic quality	1.78 (1.03)		1.91 (0.97)		1.73 (0.93)		1.72 (0.98)		0.51 (3)	.92	0.004
Hedonic quality	1.24 (1.38)		1.56 (1.26)		1.83 (0.94)		1.65 (0.96)		13.23 (3)	<.004	0.110
Overall quality	1.51 (1.13)		1.73 (1.04)		1.78 (0.82)		1.68 (0.8)		3.71 (3)	.30	0.031
Socio-emotional questionnaire											
Therapeutic relationship skills 1	34.48 (8.515)		37.63 (8.227)		35.38 (9.502)		34.53 (9.454)		4.157 (3)	.25	0.035
Therapeutic relationship skills 2	28.28 (6.548)		30.13 (6.458)		28.68 (7.388)		27.13 (5.967)		10.108 (3)	.02	0.084
Emotions in therapeutic work	11.13 (3.924)		10.85 (4.105)		10.78 (4.264)		9.8 (3.428)		5.959 (3)	.11	0.050
Virtual therapist–patient relationship											
Trust	3.55 (1.06)		3.78 (0.97)		3.72 (1.09)		3.48 (1.13)		2.27 (3)	.52	0.019
Willingness to share information	3.33 (1.07)		3.7 (1.07)		3.6 (1.13)		3.45 (1.2)		3.37 (3)	.34	0.028
Presence	5.23 (1.21)		5.18 (1.3)		5.3 (1.34)		5.22 (1.35)		0.28 (3)	.96	0.002

### Socioemotional Questionnaire and Virtual Therapist–Patient Questionnaire

While no significant differences were found among the virtual character types regarding the first and third sections of the socioemotional questionnaire (Table 3), a significant difference was observed in the second section assessing therapeutic relationship skills. Post hoc analysis revealed that the therapeutic relationship skills of the robot were significantly inferior to that of the stylized human (*z*=−2.315; *P*=.02; *P*_adj_=.02; rbb=−0.42). No significant differences were observed among the virtual character types regarding trust or willingness to share information. Additionally, presence scores did not vary significantly across the different virtual character types.

### Postquestionnaire

Participant preferences for a virtual therapist in the VRET intervention showed distinct variations. The robot was the most preferred choice (12/40, 30% of participants), followed by the stylized human (11/40, 27.5%), the cat (10/40, 25%), and finally, the realistic human (7/40, 17.5%), which was the least favored. Participants also provided diverse insights when asked about their reasons for favoring certain characters. Those who chose the realistic human mentioned its perceived “professionalism,” while those who preferred the stylized human highlighted a sense of “comfort.” However, some participants found the realistic human “uncanny.” The cat was described as having “playfully” loosened up the intervention and as appearing particularly “trusting.” The robot, valued for its “objectivity” and “anonymity,” appealed to some participants seeking a “trustworthy” therapeutic presence. Moreover, some participants also commented that its abstract nature facilitated immersion in the virtual environment and minimized distractions caused by a human character’s unnatural appearance or behavior. Regarding empathetic engagement with dog phobia, most participants (31/40, 77.5%) expressed confidence, 10% (4/40) fell in the middle, and 12.5% (5/40) expressed lower confidence.

## Discussion

### Principal Findings

This study explored how different virtual therapist embodiments were perceived within a simulated VRET scenario by a convenience sample of healthy adults. We examined the effects of different realism levels and species of virtual therapists on user experience and perceived socioemotional relationship skills. To ensure comparability among the virtual character therapist types, regardless of differing intervention scenarios, we also analyzed user experience, presence, and cybersickness across the intervention scenarios.

Our VRET application received above-average user experience scores, with no significant differences in user experience, presence, or cybersickness across the 4 intervention scenarios, which varied slightly in educational content and exposure settings. These findings allowed for the independent evaluation of the virtual character types without interference from the intervention scenarios.

Significant differences emerged in the hedonic quality of the virtual character types. The realistic human received significantly lower hedonic quality ratings than the stylized human, robot, and cat. In contrast, the cat received the highest hedonic quality ratings and scored significantly higher than both human embodiments. Contrary to our expectations, no significant differences were observed regarding trust, willingness to share information, or most socioemotional dimensions. However, the robot received lower ratings than the stylized human on 1 dimension of therapeutic relationship skills. Finally, participants’ preferences were distributed across the stylized human, robot, and cat embodiments, with the realistic human rated as the least preferred and the robot as the most preferred.

### Interpretation Within the Context of Prior Work

The most consistent finding of this study was the comparatively unfavorable evaluation of the realistic human embodiment. The realistic human received significantly lower hedonic quality ratings than all other virtual therapist embodiments and was also the least preferred character. Qualitative feedback further revealed that several participants perceived the realistic human as “uncanny.” One possible explanation for these findings is the Uncanny Valley effect [49], according to which highly human-like but imperfect artificial agents can evoke discomfort or unease. This interpretation is consistent with previous research on virtual humans and social robotics. The perception of the realistic human as more uncanny than the stylized human supports McDonnell et al [48], who found that motion anomalies in anthropomorphic virtual humans were less disturbing than in realistic virtual humans. Similarly, Volonte et al [65] reported that subtle behavioral cues were more noticeable in realistic virtual humans than in stylized ones, which may have contributed to the lower preference for the realistic human in our study. Together, these findings suggest that increasing visual realism alone does not necessarily improve users’ perceptions of virtual therapists and may even negatively affect user experience when realism is not sufficiently achieved.

Interestingly, participants responded positively to both nonhuman embodiments. The cat received the highest hedonic quality ratings, whereas the robot was the most frequently preferred virtual therapist. Qualitative responses indicated that participants often perceived the cat as playful and trustworthy, while the robot was described as objective, anonymous, and less distracting than the human embodiments. These findings are consistent with previous research suggesting that nonhuman agents can successfully fulfill social roles without adopting a human-like experience [43]. Research on animal-like agents has demonstrated that users can develop feelings of companionship and social connection with virtual pets [52,53], while research in social robotics [66,67] has shown that human-robot interactions exhibit social similarities to human-human interactions. The positive evaluations of both the cat and the robot indicate that virtual therapists do not necessarily need to resemble humans to be perceived favorably by users.

Contrary to our expectations, no significant differences were observed regarding trust or willingness to share information across the different virtual therapist embodiments. Previous research on automated VRET interventions has suggested that virtual therapists can contribute to therapeutic processes by providing psychoeducation, guidance, and supporting a relationship similar to a therapeutic alliance [27,31]. The present findings suggest that different embodiment species, including nonhuman embodiments, may be capable of supporting comparable levels of trust and willingness to share information. This interpretation is consistent with previous reviews showing that virtual therapists and ECAs have been embodied in a wide range of forms, including human avatars, photographic representations of clinicians, holographic therapists, fantasy characters, and virtual animals [32]. The positive evaluations of multiple embodiments suggest that there may not be a single universally preferred virtual therapist design. Future research should therefore investigate whether preferences for virtual therapist embodiments differ across user groups and application contexts. However, these findings should be interpreted with caution, as the relatively small convenience sample and exploratory nature of the study may have limited the ability to detect more subtle embodiment-related effects.

A similar pattern emerged in the socioemotional evaluations. Although the robot was the most frequently preferred embodiment, it received significantly lower ratings than the stylized human on 1 dimension of therapeutic relationship skills. This suggests that participants did not necessarily associate their preferred virtual therapist with the embodiment they perceived as most suitable for a therapeutic interaction. While the robot was often described as objective and anonymous, participants frequently characterized the stylized human as comforting and approachable. These findings indicate that different virtual therapist embodiments may be associated with different perceived strengths and that preference alone may not fully capture how users evaluate a virtual therapist. However, these findings should be interpreted with caution, as participants reported a relatively high affinity for technology, which may have increased the acceptance of technological embodiments such as the robot.

To our knowledge, this study is among the first to examine how realism and species influence users’ perceptions of virtual therapists within a simulated VRET setting. Previous research has demonstrated the feasibility and effectiveness of incorporating virtual therapists, coaches, or health agents into automated VRET interventions to provide psychoeducation, guidance, and user support throughout treatment [19,20,24,25]. However, little attention has been given to how different embodiments are perceived by users. The present findings therefore provide an initial indication that embodiment characteristics may influence user experience and socioemotional evaluations, while trust and willingness to share information appear less dependent on embodiment.

### Limitations

While this study provides valuable insights, several limitations must be acknowledged. First, the sample consisted of healthy young adults who were not diagnosed with dog phobia, which limits the generalizability of the findings to clinical populations.

Second, the psychoeducational dialogues with the virtual therapists lasted only approximately 3.5 minutes, which is sufficient to obtain a first impression of different embodiments but unlikely to reflect the development of therapeutic processes that typically emerge over longer interactions. As a result, constructs such as trust, willingness to share information, and perceived therapeutic relationship skills may not have been fully captured within the brief simulated encounters.

Further, since the socioemotional questionnaire was developed specifically for this study and has not undergone formal psychometric validation, its results should be interpreted with caution. Although the questionnaire’s items are based on theoretical findings and derive from constructs identified in the existing literature [56], the exploratory nature of the instrument limits the conclusions that can be drawn regarding construct validity and reliability. Future research should include formal psychometric validation procedures, such as assessments of internal consistency and construct validity, as well as comparisons with established socioemotional and therapeutic alliance measures.

Furthermore, the study relied on qualitative feedback to assess the Uncanny Valley effect, which limits the precision of these findings. Finally, while the Wizard-of-Oz approach increased experimental control and consistency across conditions, it may have reduced the ecological validity of therapist-participant interactions compared with fully autonomous conversational systems.

### Conclusions

Despite the potential of VRET for treating specific phobias, it is not yet widely integrated into clinical practice. This underscores the need for further research into its key components, such as the role of virtual therapists, to improve its implementation and accessibility for individuals with specific phobias. This study represents an initial exploration of how the realism and species of virtual therapist character types may influence user experience and their socioemotional evaluation in a VRET application. The realistic human model received lower hedonic quality ratings and preference scores than the other embodiments, suggesting that increased visual realism does not necessarily result in more positive perceptions of virtual therapists and may, in some cases, lead to less favorable user responses. Additionally, the cat model’s higher hedonic quality and the robot’s popularity suggest that different user groups may respond differently to virtual therapist embodiments. Taken together, the findings indicate that virtual therapist design may influence user experience and socioemotional perceptions, highlighting the importance of considering embodiment characteristics when developing automated VRET interventions. Further research is needed to better understand the impact of virtual therapist design in VRET applications. Future studies should compare realistic and stylized virtual characters, as well as different species, across diverse user groups, longer interaction periods, and clinical settings.

## Abbreviations

ATI: Affinity for Technology Interaction
ECA: embodied conversational agent
UEQ-S: User Experience Questionnaire Short Version
VR: virtual reality
VRET: virtual reality exposure therapy

## References

[1] Wardenaar KJ, Lim CCW, Al-Hamzawi AO (2017). The cross-national epidemiology of specific phobia in the World Mental Health Surveys. Psychol Med.

[2] Rachman S (1972). Verhaltenstherapie Bei Phobien [Behavior Therapy for Phobias].

[3] Steinmann SA, Wootton BM, Tolin DF, Friedman HS (2016). Encyclopedia of Mental Health.

[4] Leibetseder M (2018). Grundlagenbuch Verhaltenstherapie: Diagnostik, Methoden, Anwendungsbereiche, Sprachanalysen [Fundamentals of Behavior Therapy: Diagnostics, Methods, Areas of Application, Language Analyses].

[5] Hamm A (2006). Fortschritte Der Psychotherapie Manuale Für Die Praxis.

[6] Schröder D, Wrona KJ, Müller F, Heinemann S, Fischer F, Dockweiler C (2023). Impact of virtual reality applications in the treatment of anxiety disorders: a systematic review and meta-analysis of randomized-controlled trials. J Behav Ther Exp Psychiatry.

[7] Mackenzie CS, Reynolds K, Cairney J, Streiner DL, Sareen J (2012). Disorder-specific mental health service use for mood and anxiety disorders: associations with age, sex, and psychiatric comorbidity. Depress Anxiety.

[8] Rizzo AS, Bouchard S (2019). Virtual Reality for Psychological and Neurocognitive Interventions.

[9] Boeldt D, McMahon E, McFaul M, Greenleaf W (2019). Using virtual reality exposure therapy to enhance treatment of anxiety disorders: identifying areas of clinical adoption and potential obstacles. Front Psychiatry.

[10] Slater M, Sanchez-Vives MV (2016). Enhancing our lives with immersive virtual reality. Front Robot AI.

[11] Slater M (2018). Immersion and the illusion of presence in virtual reality. Br J Psychol.

[12] Emmelkamp PMG (2005). Technological innovations in clinical assessment and psychotherapy. Psychother Psychosom.

[13] Garcia-Palacios A, Botella C, Hoffman H, Fabregat S (2007). Comparing acceptance and refusal rates of virtual reality exposure vs. in vivo exposure by patients with specific phobias. Cyberpsychol Behav.

[14] Garcia-Palacios A, Hoffman HG, See SK, Tsai A, Botella C (2001). Redefining therapeutic success with virtual reality exposure therapy. Cyberpsychol Behav.

[15] Powers MB, Emmelkamp PMG (2008). Virtual reality exposure therapy for anxiety disorders: a meta-analysis. J Anxiety Disord.

[16] Morina N, Ijntema H, Meyerbröker K, Emmelkamp PMG (2015). Can virtual reality exposure therapy gains be generalized to real-life? A meta-analysis of studies applying behavioral assessments. Behav Res Ther.

[17] Carl E, Stein AT, Levihn-Coon A (2019). Virtual reality exposure therapy for anxiety and related disorders: a meta-analysis of randomized controlled trials. J Anxiety Disord.

[18] Kazdin AE, Blase SL (2011). Rebooting psychotherapy research and practice to reduce the burden of mental illness. Perspect Psychol Sci.

[19] Freeman D, Haselton P, Freeman J (2018). Automated psychological therapy using immersive virtual reality for treatment of fear of heights: a single-blind, parallel-group, randomised controlled trial. Lancet Psychiatry.

[20] Lindner P, Miloff A, Bergman C, Andersson G, Hamilton W, Carlbring P (2020). Gamified, automated virtual reality exposure therapy for fear of spiders: a single-subject trial under simulated real-world conditions. Front Psychiatry.

[21] Baumeister H, Reichler L, Munzinger M, Lin J (2014). The impact of guidance on Internet-based mental health interventions—a systematic review. Internet Interv.

[22] Fehribach JR, Toffolo MBJ, Cornelisz I (2021). Virtual reality self-help treatment for aviophobia: protocol for a randomized controlled trial. JMIR Res Protoc.

[23] González-Lozoya SM, Meza-Kubo V, Dominguez-Rodriguez A, Ramírez-Fernández C, Morán AL (2021). A self-applied, virtual therapist assisted-based “thera” treatment for small animal phobia in Mexican population: a preliminary evaluation.

[24] Donker T, Cornelisz I, van Klaveren C (2019). Effectiveness of self-guided app-based virtual reality cognitive behavior therapy for acrophobia: a randomized clinical trial. JAMA Psychiatry.

[25] Miloff A, Lindner P, Dafgård P (2019). Automated virtual reality exposure therapy for spider phobia vs. in-vivo one-session treatment: a randomized non-inferiority trial. Behav Res Ther.

[26] Lindner P, Rozental A, Jurell A (2020). Experiences of gamified and automated virtual reality exposure therapy for spider phobia: qualitative study. JMIR Serious Games.

[27] Miloff A, Carlbring P, Hamilton W, Andersson G, Reuterskiöld L, Lindner P (2020). Measuring alliance toward embodied virtual therapists in the era of automated treatments with the Virtual Therapist Alliance Scale (VTAS): development and psychometric evaluation. J Med Internet Res.

[28] Wei S, Freeman D, Rovira A (2023). A randomised controlled test of emotional attributes of a virtual coach within a virtual reality (VR) mental health treatment. Sci Rep.

[29] Wei S, Freeman D, Rovira A (2025). Virtual humans in virtual reality mental health research: systematic review. JMIR XR Spat Comput.

[30] Cassell J, Bickmore T, Campbell L, Vilhjálmsson H, Yan H, Cassell J, Sullivan J, Prevost S, Churchill EF (2000). Embodied Conversational Agents.

[31] Lindner P (2021). Better, virtually: the past, present, and future of virtual reality cognitive behavior therapy. J Cogn Ther.

[32] Mitruț O, Moldoveanu A, Petrescu L, Petrescu C, Moldoveanu F, Chen JYC, Fragomeni G A review of virtual therapists in anxiety and phobias alleviating applications.

[33] Lilliengren P, Falkenström F, Sandell R, Mothander PR, Werbart A (2015). Secure attachment to therapist, alliance, and outcome in psychoanalytic psychotherapy with young adults. J Couns Psychol.

[34] Pan X, Banakou D, Slater M, D’Mello S, Graesser A, Schuller B, Martin JC (2011). Affective Computing and Intelligent Interaction ACII 2011.

[35] Robb A, Kopper R, Ambani R (2013). Leveraging virtual humans to effectively prepare learners for stressful interpersonal experiences. IEEE Trans Vis Comput Graph.

[36] Oh CS, Bailenson JN, Welch GF (2018). A systematic review of social presence: definition, antecedents, and implications. Front Robot AI.

[37] Wienrich C, Döllinger N, Hein R (2021). Behavioral Framework of Immersive Technologies (BehaveFIT): how and why virtual reality can support behavioral change processes. Front Virtual Real.

[38] Biocca F, Harms C, Burgoon JK (2003). Toward a more robust theory and measure of social presence: review and suggested criteria. Presence Teleoperators Virtual Environ.

[39] Kort YAW, IJsselsteijn WA, Poels K (2007). Digital games as social presence technology: development of the social presence in gaming questionnaire (SPGQ). https://research.tue.nl/en/publications/digital-games-as-social-presence-technology-development-of-the-so/.

[40] You C, Ghosh R, Maxim A, Stuart J, Cooks E, Lok B How does a virtual human earn your trust? guidelines to improve willingness to self-disclose to intelligent virtual agents.

[41] Horvath AO (2018). Research on the alliance: Knowledge in search of a theory. Psychother Res.

[42] Provoost S, Lau HM, Ruwaard J, Riper H (2017). Embodied conversational agents in clinical psychology: a scoping review. J Med Internet Res.

[43] Straßmann C, Krämer NC, Beskow J, Peters C, Castellano G, O’Sullivan C, Leite I, Kopp S (2017). Intell Virtual Agents.

[44] Kyrlitsias C, Michael-Grigoriou D (2022). Social interaction with agents and avatars in immersive virtual environments: a survey. Front Virtual Real.

[45] Fong T, Nourbakhsh I, Dautenhahn K (2003). A survey of socially interactive robots. Robot Auton Syst.

[46] Yang FC, Acevedo P, Guo S, Choi M, Mousas C (2025). Embodied conversational agents in extended reality: a systematic review. IEEE Access.

[47] ter Stal S, Kramer LL, Tabak M, op den Akker H, Hermens H (2020). Design features of embodied conversational agents in eHealth: a literature review. Int J Hum Comput Stud.

[48] McDonnell R, Breidt M, Bülthoff HH (2012). Render me real? Investigating the effect of render style on the perception of animated virtual humans. ACM Trans Graph.

[49] Mori M, MacDorman KF, Kageki N (2012). The uncanny valley. IEEE Robot Autom Mag.

[50] Stein C, Lee N, Lee N (2018). Encyclopedia of Computer Graphics and Games.

[51] Chi NC, Sparks O, Lin SY, Lazar A, Thompson HJ, Demiris G (2017). Pilot testing a digital pet avatar for older adults. Geriatr Nurs.

[52] Bott N, Wexler S, Drury L (2019). A protocol-driven, bedside digital conversational agent to support nurse teams and mitigate risks of hospitalization in older adults: case control pre-post study. J Med Internet Res.

[53] Schwind V, Leicht K, Jäger S, Wolf K, Henze N (2018). Is there an uncanny valley of virtual animals? A quantitative and qualitative investigation. Int J Hum Comput Stud.

[54] Sierra Rativa A, Postma M, van Zaanen M (2022). The uncanny valley of a virtual animal. Comput Animation Virtual.

[55] Dahlbäck N, Jönsson A, Ahrenberg L (1993). Wizard of Oz studies—why and how. Knowl Based Syst.

[56] Heinonen E, Nissen-Lie HA (2020). The professional and personal characteristics of effective psychotherapists: a systematic review. Psychother Res.

[57] Orlinsky DE, Rønnestad MH, Collaborative Research Network of the Society for Psychotherapy Research (2005). How Psychotherapists Develop: A Study of Therapeutic Work and Professional Growth.

[58] DeVault D, Artstein R, Benn G SimSensei kiosk: a virtual human interviewer for healthcare decision support.

[59] Rheu M, Shin JY, Peng W, Huh-Yoo J (2021). Systematic review: trust-building factors and implications for conversational agent design. Int J Hum Comput Interact.

[60] Schrepp M, Hinderks A, Thomaschewski J, Marcus A, Marcus A (2014). Design, User Experience, and Usability Theories, Methods, and Tools for Designing the User Experience.

[61] Franke T, Attig C, Wessel D (2019). A personal resource for technology interaction: development and validation of the Affinity for Technology Interaction (ATI) Scale. Int J Hum Comput Interact.

[62] Arnfred B, Svendsen JK, Adjourlu A, Horthøj C (2023). Scoping review of the hardware and software features of virtual reality exposure therapy for social anxiety disorder, agoraphobia, and specific phobia. Front Virtual Real.

[63] Schubert T, Friedmann F, Regenbrecht H (2001). The experience of presence: factor analytic insights. Presence Teleoperators Virtual Environ.

[64] Dörner R, Broll W, Grimm P, Jung B (2019). Virtual Und Augmented Reality (VR/AR).

[65] Volonte M, Babu SV, Chaturvedi H (2016). Effects of virtual human appearance fidelity on emotion contagion in affective inter-personal simulations. IEEE Trans Visual Comput Graphics.

[66] Hoffmann L (2017). That robot touch that means so much: on the psychological effects of human-robot touch [doctoral dissertation]. https://duepublico2.uni-due.de/receive/duepublico_mods_00043331.

[67] Hoffmann L, Krämer NC (2021). The persuasive power of robot touch. Behavioral and evaluative consequences of non-functional touch from a robot. PLoS One.

